# The possible meaning of fractional anisotropy measurement of the cervical spinal cord in correct diagnosis of amyotrophic lateral sclerosis

**DOI:** 10.1007/s10072-015-2418-4

**Published:** 2015-11-21

**Authors:** Slawomir Budrewicz, Pawel Szewczyk, Joanna Bladowska, Ryszard Podemski, Ewa Koziorowska-Gawron, Maria Ejma, Krzysztof Słotwiński, Magdalena Koszewicz

**Affiliations:** Department of Neurology, Wroclaw Medical University, Borowska 213, 50-556 Wroclaw, Poland; Department of General Radiology, Interventional Radiology and Neuroradiology, Wroclaw Medical University, Wroclaw, Poland

**Keywords:** Amyotrophic lateral sclerosis, Motor pathway, Cervical spinal cord, Neuroimaging, Fractional anisotropy, Diffusion tensor imaging

## Abstract

Diagnosis of amyotrophic lateral sclerosis (ALS) is based on clinical criteria and electrophysiological tests (electromyography, and transcranial magnetic stimulation). In the search for ALS biomarkers, the role of imaging procedures is currently emphasized, especially modern MR techniques. MR procedures were performed on 15 ALS patients and a sex- and age-matched control group. The MR examinations were performed with a 1.5-T MR unit, and the protocol consisted of sagittal T1-weighed images, sagittal and axial T2-weighed images, and sagittal T2-weighed FAT SAT images followed by an axial diffusion tensor imaging (DTI) sequence of the cervical spinal cord. FA values in individual segments of the cervical spinal cord were decreased in the ALS group in comparison with the control group. After comparing FA values for anterior, posterior, and lateral corticospinal columns, the greatest difference was observed between the C2 and C5 segments. Spinal cord assessment with the use of FA measurements allows for confirmation of the motor pathways lesion in ALS patients. The method, together with clinical criteria, could be helpful in ALS diagnosis, assessment of clinical course, or even the effects of new drugs. The results also confirmed the theory of the generalized character of ALS.

## Introduction

Amyotrophic lateral sclerosis (ALS) is a degenerative nervous system disease of unknown etiology, with lower and upper motor neuron lesion, following neuronal loss and gliosis [1, 2]. The incidence rate of the disease is 1–2 cases per 100,000 per year. The diagnosis is mostly based on clinical criteria (El Escorial with Awaji modifications) and electrophysiological tests (EMG, and transcranial magnetic stimulation). Clinical assessment provides an accuracy level of about 95 %, although this has not been unequivocally confirmed in clinical tests. ALS diagnosis, especially at the early stage of the disease, can be difficult. EMG tests, with an assessment of the dynamics of electromyographic changes in subsequent studies, together with transcranial magnetic stimulation, may prove helpful. In recent years, neuroimaging techniques, especially modern MR techniques, have been considered increasingly important [1, 2]. ALS diagnosis may incorporate MR proton spectroscopy and imaging based on perfusion techniques, magnetization transfer of the brain, and occasionally studies of the spinal cord [3–10].

The aim of the study was to assess the possible meaning and usefulness of fractional anisotropy (FA) measurements of the cervical spinal cord in the confirmation of unequivocal ALS diagnosis, primarily based on the Awaji criteria.

## Materials and methods

Fifteen patients with ALS (5 definite, 3 probable, and 7 possible diagnoses according to Awai criteria), 5 males and 10 females (mean age—53.7 years), were enrolled in this study. Mean duration of ALS symptoms was 7.3 months. All patients were subject to clinical examination and EMG (motor and sensory conduction tests in two nerves, electromyography in 4 muscles). The control group consisted of 15 healthy individuals, sex- and age-matched.

This study was conducted in accordance with the guidelines of the local University Ethics Committee for conducting research involving humans. Each patient signed his/her informed consent before participation in the examination.

The MR examinations were performed with a 1.5-T MR unit (Signa Hdx, GE Medical Systems) with a 33mT/m maximum gradient strength, using a sixteen-channel coil dedicated for head and spine imaging. The MR protocol consisted of sagittal T1-weighed images (TR/TE 555/10 ms), sagittal and axial T2-weighed images (TR/TE 3580/111 ms), and sagittal T2-weighed FAT SAT images (TR/TE 3380/118 ms), followed by an axial DTI sequence.

DTI acquisition was based on single-shot spin-echo echo-planar imaging (SE/EPI) with the following parameters: TR/TE 10.000/100 ms, a 160 × 160 mm field of view, matrix 96 × 96, in-plane image resolution 1.6 × 1.6 mm with 4-mm-thick axial slices parallel to the intervertebral disk space, no gap, and number of acquisitions: 2. In each study, the examination frame was adjusted to cover the length of the spinal cord from the first to fifth cervical vertebrae. Diffusion was measured with two *b* values, 0 and 1000 mm^2^/s resulting in one image without and 14 with diffusion weighting. The DTI acquisition time ranged from 5 to 7 min. Patients and volunteers were asked to breathe very slowly and to avoid swallowing during DTI acquisition.

**Fig. 1:**
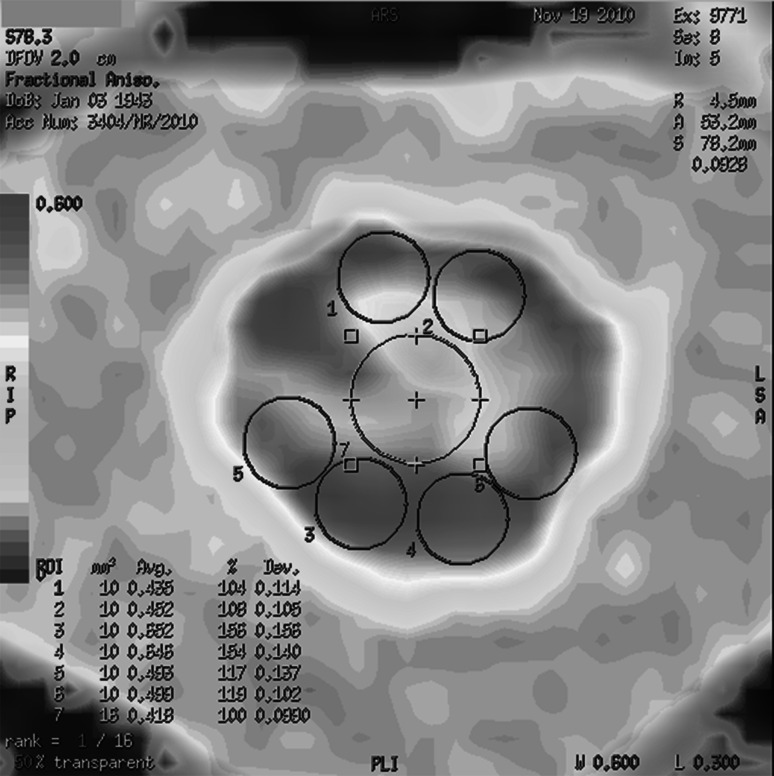
Diffusion tensor imaging study. FA parametric map shows a transverse section of the spinal cord on the C1 level. FA values were measured using small, *circular regions* of interest (ROIs) drawn within the anterior (*purple circles number 1*, *2*), posterior (*purple circles 3, 4*), and lateral (*purple circles 5*, *6*) spinal cord columns (each ROI size 10 mm^2^) and within the *central gray matter* (*green circular* ROI size 16 mm^2^—*number 7*)

Image post-processing was performed using the Functool software (GE ADW 4.4 workstation). Fractional anisotropy (FA) transverse maps were generated and FA values were measured at levels C1 to C5 using small, circular regions of interest. Regions of interest (ROIs) sized 10 mm^2^ were drawn within the anterior, lateral, and posterior columns of the spinal cord, according to the most accurate axial B0 image, as shown in Fig. 1. Additionally, a ROI (size 16 mm^2^) was drawn over the central region of the spinal cord in order to measure the FA value within the gray matter (Fig. 1). Special attention was paid to avoiding partial volume effects, magnetic susceptibility effects, and motion artifacts.

In a fractional anisotropy test, the values for each type of column were summarized on the right and on the left, and then the values for each individual segment of the cervical spinal cord from C2 to C5 were compared.

The results were subject to statistical analysis. Mean values (*X*), median values (*M*), ranges (min–max), and standard deviations (SD) of continuous parameters were calculated. The hypothesis about the equality of mean parameters in given groups was verified with the ANOVA variance analysis method or, in the case of groups comprising innumerous cases or heterogeneous variance, with the non-parametric Kruskal–Wallis method of analysis by ranks. Homogeneity of variance was verified with Bartlett’s test. For chosen pairs of parameters, correlation analysis was carried out by calculating the Spearman’s correlation coefficient (*R*). A value of *P* ≤ 0.05 was considered to be statistically significant. The statistical analysis was carried out with the EPIINFO Ver. 3.5.2 software package (17-12-2010 version).

## Results

**Fig. 2:**
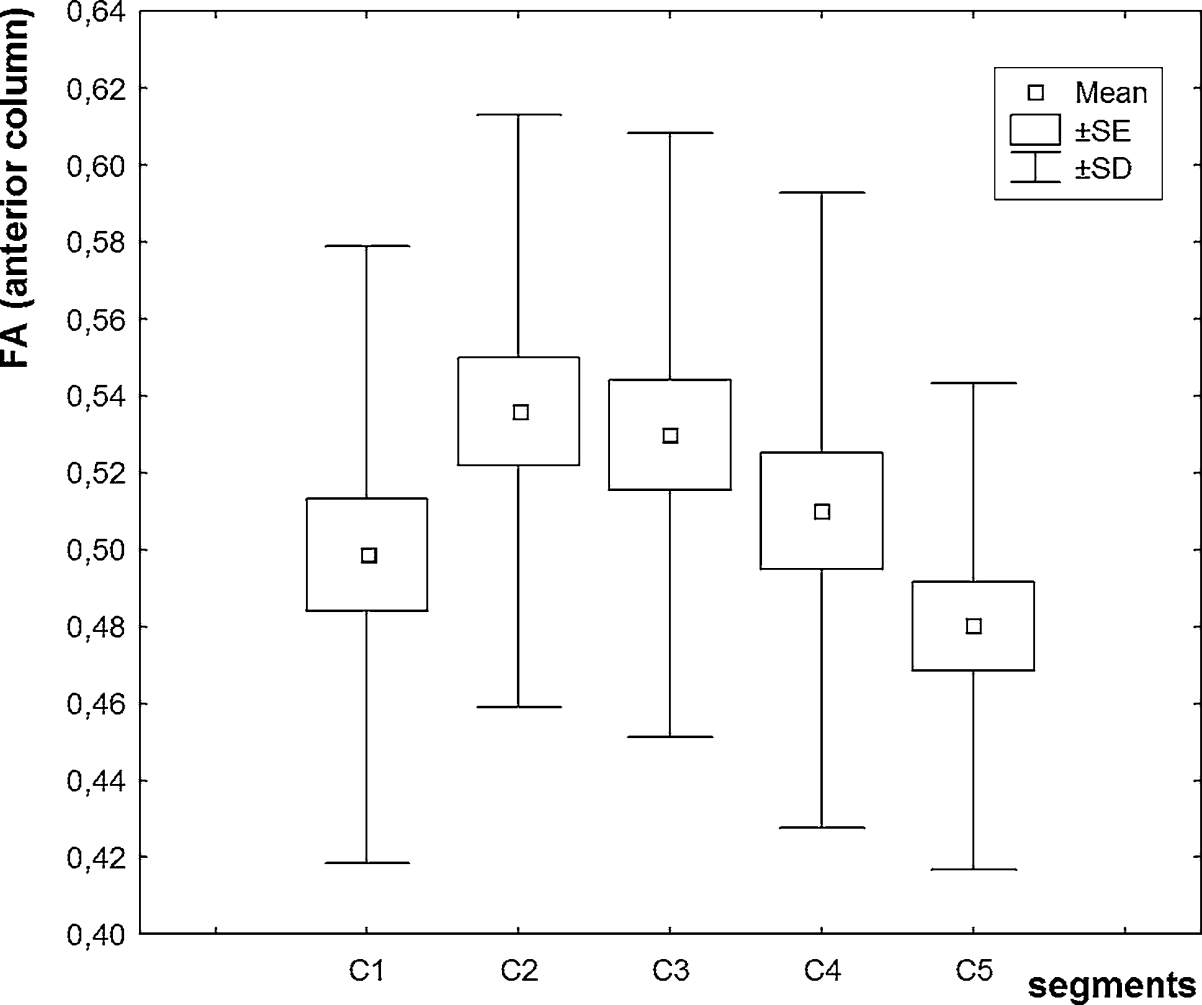
Diagram of the values of fractional anisotropy in cervical cord segments (C1–C5) (*anterior column* of spinal cord)

**Fig. 3:**
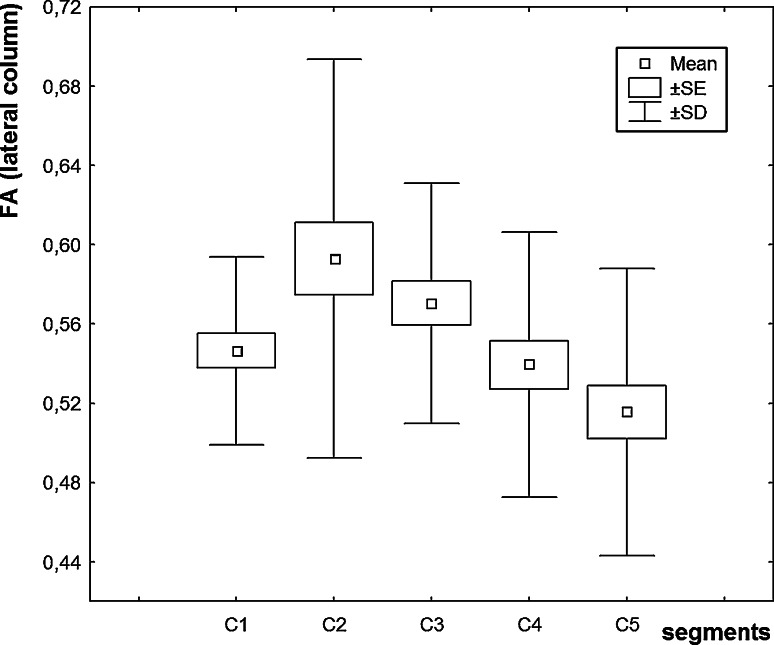
Diagram of the values of fractional anisotropy in cervical cord segments (C1–C5) (*lateral column* of spinal cord)

**Fig. 4:**
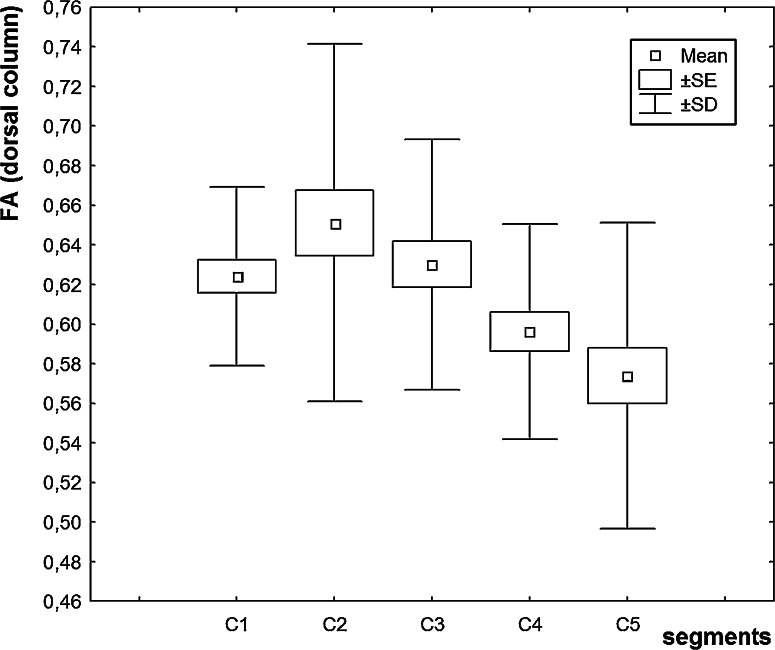
Diagram of the values of fractional anisotropy in cervical cord segments (C1–C5) (*posterior column* of spinal cord)

FA values in the individual segments of the anterior, lateral, and posterior columns and in the central region of cervical spinal cord were diminished in ALS patients in comparison to the control group (Table 1). In the patients’ group, we revealed a statistically significant decline in FA values in all the columns and in the consecutive segments, e.g., between C2 and C3, and C3 and C5 segments (Figs 2, 3, 4). In the central region, FA values were significantly diminished between C1 and other cervical segments, maximally between C1 and C3. We did not observe any lowering of FA values in the consecutive segments in that region. In the patients’ group, FA values were significantly decreased in the consecutive cervical spinal cord segments: in the anterior column between C2 and C3, posterior column between C3 and C5, and lateral columns between levels C2 and C4, C2 and C5, C3 and C4, and C3 and C5. In patients with definite ALS recognition, we revealed lower values for the FA parameter than those were seen in the group of the probable recognition (Table 2). According to our own experiences, we assumed that the values obtained from the first segment (C1) do not have a good diagnostic value because of its anatomically transient character between bulb and spinal cord, and ROI estimation is very difficult in this region.

**Table 1:** Mean, maximal, minimal, and standard deviation values of FA in circular regions of interest (ROIs) in anterior column (AC), posterior column (PC), lateral column (LC), and central region (CR) in the control, and patients’ groups

Group	Controls (*n* = 10)					Patients (*n* = 15)					*p*
	*M*	Min	Max	25Q	75Q	*M*	Min	Max	25Q	75Q	
Age	30.0	23.0	45.0	27.0	37.0	58.0	21.0	75.0	33.0	60.0	0.0055
Sex W/M	6/4					10/5					1.0F

Group	Controls (*n* = 50)				Patients (*n* = 75)				*P*
	*X*	SD	Min	Max	*X*	SD	Min	Max	
AC ROI R	0.611	0.066	0.454	0.754	0.512	0.078	0.354	0.695	0.00000
AC ROI L	0.602	0.072	0.426	0.787	0.510	0.079	0.349	0.752	0.00000
PC ROI R	0.678	0.069	0.563	0.854	0.614	0.076	0.371	0.791	0.00001
PC ROI L	0.692	0.076	0.555	0.905	0.616	0.069	0.437	0.776	0.00000
LC ROI R	0.660	0.072	0.490	0.821	0.554	0.078	0.400	0.793	0.00000
LC ROI L	0.660	0.088	0.456	0.881	0.552	0.073	0.431	0.823	0.00000
CR ROI	0.618	0.070	0.435	0.798	0.551	0.069	0.376	0.711	0.00000

**Table 2:** Mean, maximal, minimal, and standard deviation values of FA in circular regions of interest (ROIs) in anterior column (AC), posterior column (PC), lateral column (LC), and central region (CR) in definite and possible ALS patients’ groups

Group	Definite ALS								Possible ALS								*P*
	*X*	*N*	SD	Min	Max	25Q	*M*	75Q	*X*	*N*	SD	Min	Max	25Q	*M*	75Q	
AC ROI R	0.507	45	0.084	0.354	0.695	0.444	0.498	0.567	0.530	25	0.069	0.368	0.662	0.488	0.537	0.579	0.150
AC ROI L	0.501	45	0.076	0.349	0.640	0.436	0.517	0.558	0.532	25	0.086	0.372	0.752	0.474	0.523	0.586	0.237
PC ROI R	0.593	45	0.076	0.371	0.767	0.551	0.583	0.643	0.646	25	0.062	0.522	0.791	0.615	0.646	0.670	0.0037
PC ROI L	0.600	45	0.074	0.437	0.776	0.552	0.587	0.641	0.641	25	0.054	0.556	0.775	0.602	0.628	0.671	0.0150
LC ROI R	0.547	45	0.078	0.410	0.793	0.485	0.556	0.579	0.570	25	0.075	0.431	0.699	0.524	0.575	0.629	0.186
LC ROI L	0.549	45	0.081	0.431	0.823	0.492	0.535	0.588	0.561	25	0.064	0.473	0.729	0.513	0.549	0.608	0.295
CR ROI	0.537	45	0.067	0.376	0.655	0.493	0.531	0.582	0.579	25	0.066	0.461	0.711	0.539	0.582	0.629	0.0219

## Discussion

DTI is a non-invasive MR technique, useful in visualizing long white matter columns and assessing their integrity. This technique allows for a quantitative assessment of the free diffusion of water particles in the extracellular space, expressed as an apparent diffusion coefficient, and a qualitative and quantitative assessment of the directivity of diffusion. In the brain and spinal cord white matter, the movement of water particles is limited and does not present an isotropic nature (equal occurrence in all directions). The presence of natural barriers to diffusion, such as cellular membranes and myelin sheaths, is responsible for this phenomenon. The DTI method is sensitive in detecting changes in the microstructure of tissues; therefore, it can be used as a sensitive diagnostic tool in neuro-degenerative diseases. Limiting diffusion in directions that are perpendicular to the white matter fibers, with a relative preponderance of diffusion aligned with axon columns, is referred to as diffusion anisotropy. The degree of anisotropy can be assessed using the parameter of FA. FA is defined as a non-specific indicator of white matter integrity and its decrease is consistent with the degradation or impairment of white matter coherence due to microstructural pathology [12].

Hitherto, research aimed at developing visual ALS biomarkers has focused mainly on brain testing and has neglected the significance of the spinal cord [4, 8], although the role of spinal cord lesion in the course of this disease has been confirmed in anatomo-pathological examinations. Additionally, imaging of the spinal cord is technically difficult [3], and the literature regarding the meaning of diagnostic imaging of the spinal cord in ALS is relatively scarce [6, 7, 12]. Nair et al. [3] indicated the significant role of spinal cord DTI in ALS diagnostics. The authors compared the results of brain stem and cervical spinal cord DTI with limb function tests. They reported a significant decrease in the FA value within the cervical spinal cord white matter in the ALS group, but in this study, ROIs were drawn over the entire white matter of the spinal cord. Underwood et al. [13], in an animal model, showed axonal lumbar spinal cord damage revealed via a DTI technique. FA values were reduced only in the area of the anterior white matter and only in motor columns, excluding sensory pathways. The decrease in FA values was correlated with disease progression. They hypothesized that decreased FA values most likely reflect axon number and demyelination. Therefore, DTI may be used as a useful non-invasive technique in the assessment and monitoring of the progression of ALS.

In our study, FA values were assessed using small, circular ROIs drawn in anterior, lateral, and posterior corticospinal columns in consecutive cervical segments. The values for individual ROIs were bilaterally determined on one level of the cervical segment. Our methodology was different from the one proposed by Nair et al. [3] and allowed for a more accurate designation of the FA parameter in corticospinal columns and the central area of the spinal cord.

The decrease in FA values in consecutive segments of the cervical spinal cord may indicate the loss of neurons and accompanying gliosis, and may be used as a marker of spinal cord degenerative processes in ALS. In animal models, there were no changes of FA values within sensory pathways [13]. In our study, FA changes were observed in posterior columns responsible for carrying proprioceptive impulses. The question about the possibility of the coexistence of degenerative changes in sensory columns remains open, although nowadays ALS is thought to be a generalized disease, affecting not only motor pathways.

The decrease in FA values in consecutive cervical segments with a caudal predominance, as observed in our study, could support the “dying back” theory. The degenerative changes in ALS might be treated as a distal axonopathy [2, 14]. Decreased FA values in a caudal direction were observed not only in the anterior and lateral but also in the posterior columns. The statistically significant differences between averaged FA values in all spinal cord columns and in the central region might indicate disturbances of axonal integrity not only in the motor pathway in patients with ALS.

## Conclusions

Spinal cord assessment with the use of FA measurements allows for the confirmation of the lesion of motor pathways in ALS patients. The method, together with clinical criteria, could be helpful in ALS diagnosis, assessment of clinical course, or even the effects of new drugs. The results also confirmed the theory of the generalized character of ALS.
